# The role of bacteriophages and CRISPR-Cas in combating multidrug-resistant bacteria

**DOI:** 10.1007/s13659-025-00567-y

**Published:** 2026-01-10

**Authors:** Ciamak Ghazaei

**Affiliations:** University of Mohaghegh Ardabili, Ardabil, Islamic Republic of Iran

**Keywords:** Bacteria, CRISPR-Cas, Pathogens, Antimicrobial resistance, Infectious diseases

## Abstract

The alarming increase of multidrug-resistant (MDR) bacteria presents a serious global health crisis, reducing the effectivenessof traditional antibiotics and requiring alternative therapeutic strategies. Among the most promising innovations are bacteriophages—viruses that specifically infect bacteria—and CRISPR-Cas systems, molecular tools enabling precise genome editing. These technologies individually offer targeted antibacterial activity with minimal disturbance to the host microbiota. When combined, they forma synergistic platform capable of overcoming many limitations of conventional antibiotics, including broad-spectrum activity, resistance development, and limited adaptability. This review examinesmechanisms of bacterial resistance, the biological foundation of bacteriophages and CRISPR-Cas systems, and their application in fighting MDR pathogens. However, significant challenges remain, including delivery barriers, off-target effects, regulatory uncertainty, and public acceptance of gene-editing tools. Antimicrobial resistance now tanks among the top threats to global health, with an estimated burden exceeding one million deaths annually, surpassing many other infectious diseases. The article concludes with a discussion of the clinical prospects of phage-CRISPR therapies and highlights key areas for future research. By merging the specificity of phages with the programmable strength of CRISPR, these biotechnological advances provide a powerful and approach to address the growing threat of antibiotic resistance.

**Graphical Abstract:**

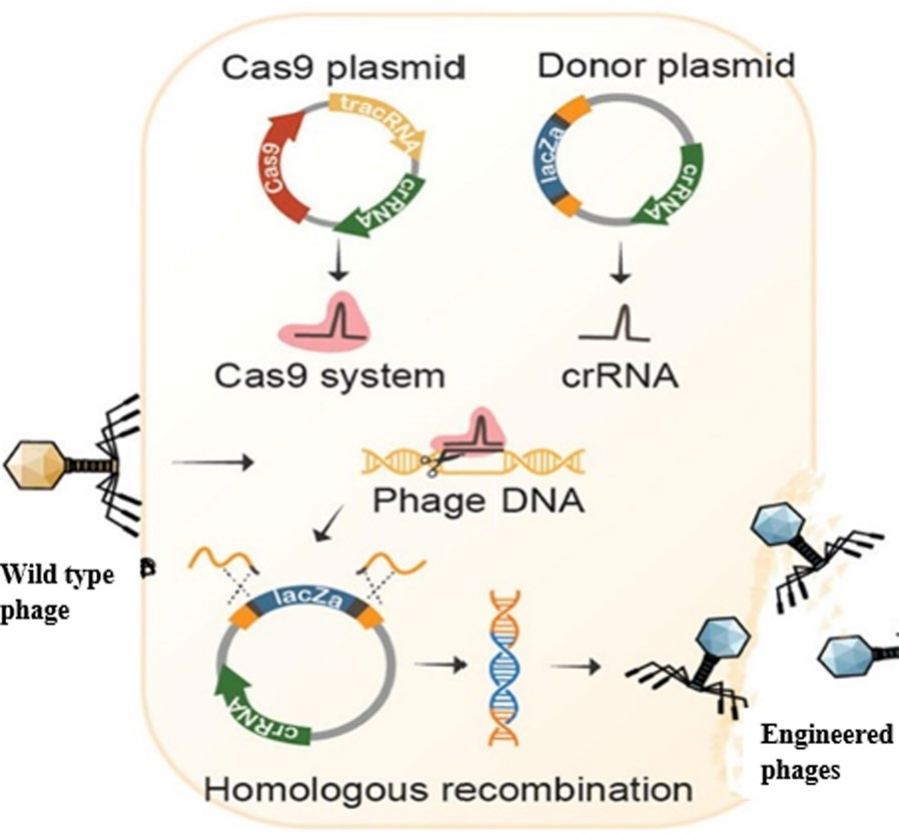

## Introduction

The rise of multidrug-resistant (MDR) bacteria has become one of the biggestthreats to global health in the twenty-first century. MDR bacteria are typically defined as pathogens resistant to at least one agent in at least three antimicrobial categories [1]. MDR pathogens have gained resistance to multiple antibiotic classes, undoing decades of progress in fighting bacterial infections and leading to longer hospital stays, higher treatment costs, and increased mortality. The World Health Organization (WHO) considers antimicrobial resistance one of the top ten global public health threats, warning of a post-antibiotic era in which even minor infections could become deadly. In 2019 alone, antimicrobial resistance was linked to around 4.95 million deaths globally, highlighting its severe impact on public health [2]. Drug-resistant bacteria cause 1.27 million deaths each year, surpassing HIV and malaria combined [3]. Many of these deaths mainly concentrated in low- and middle-income countries, especially among children under five. Some estimates suggest that the annual death toll could reach 10 million by 2050 [4].

Focusing on the most threatening Gram-negative bacteria, the WHO published a list of antibiotic-resistant pathogens. The list categorizes these infections into three priority levels—medium, high, and critical -based on the urgency of developing new antibiotics. *Pseudomonas aeruginosa*, *Acinetobacter baumannii*, and *Enterobacteriaceae* are among the main groups of MDR bacteria that can cause bloodstream infections and other serious illnesses in hospitalized patients, including pneumonia. Common diseases such as gonorrhea and food poisoning are caused by bacteria such as *Salmonella*, which are categorized in the high and medium priority groups [5–9]. According to a WHO priority list, pathogens like *carbapenem-resistant Enterobacteriaceae* (CRE) and *methicillin-resistant Staphylococcus aureus* (MRSA) are considered critical threats [10]. Bacteria evade these drugs through various mechanisms, as discussed in Table 1.

**Table 1:** Key mechanisms of antibiotic resistance with representative examples

Resistance mechanism	Description	Example	References
Drug inactivation	Bacteria deactivate antibiotics using protective enzymes, e.g., Β-lactamases	Penicillin G inactivation by β-lactamase; *Pseudomonas* resistance via β-lactamases inactivating the amide bond	[11–14]
Target alteration	Structural changes to antibiotic target sites, often via protective proteins or mutations, interfere with drug binding	MRSA inactivates antibiotics by binding protective proteins; Gentamycin inactivates antibiotics (Aminoglycoside) through interference with translation	[11–15]
Drug efflux	Increase efflux pumps activity or decrease drug permeability, leading to low antibiotic levels and increased susceptibility	Fluoroquinolone resistance via efflux pumps; Less significant for Gram-positive bacteria (lack lipopolysaccharides in outer membrane)	[13–15]
Reduced uptake/permeability	Alteration of outer membrane porins, increased hydrophobicity and decreased permeability	Resistance of *P. Aeruginosa* throughmutations of porin-associated genes	[11–14]

Currently, two main approaches are used to treat MDR: pathogen-directed and host-directed strategies. Pathogen-directed treatments aims to lower bacterial toxicity through methods like monoclonal antibodies targeting specific antigens, disrupting adhesion or biofilms, neutralising virulence factors, re-expressing virulence genes for immune clearance, interfering with quorum sensing (QS) using compounds like 6-gingerol along with antibiotics, sequestering or competing with toxins for their receptor binding, and modifying bacterial gene expression with agents like virstatin and regacin to reduce pilus formation and motility. Recent research shows increasing resistance of *P. aeruginosa* to tobramycin; however, 6-gingerol from ginger has shown potential to inhibit QS. Combining 6-gingerol derivatives with tobramycin more effectively suppresses *P. aeruginosa’s* virulence factors including QS and biofilm formation compared to individual treatments. Host-directed therapies focus on boosting immune responses by increasing phagocyte activity, modulating cytokines and chemokines, and utilising lipid mediators, such as leukotriene B4, to generate reactive oxygen species. Other strategies aim to improve phagocyte functions, increase Toll-like receptor signalling to increase cytokine output, and repurposing existing drugs, such as statins, to reduce mortality, sepsis, and pneumonia. Tamoxifen is also used to prevent MRSA infections. Notably, when macrophages are treated with leukotriene B4 (LTB4), they produce reactive oxygen species via the NADPH oxidase, aiding in MDR control [16].

One approach to address this risky situation is to develop new antibacterial agents [17]. Currently, nearly 43 new antibiotics, including lascufloxacin, apramycin (EBL-10031), cefiderocol, contezolid acefosamil, and colithromycin, are in development by leading pharmaceutical companies, demonstrating strong efficacy against severe bacterial infections [18]. Of these, 18 target gram-negative resistant pathogens such as *Enterobacteriaceae* and *P. aeruginosa*, while 10 target resistant *Neisseria gonorrhoeae*. Some have FDA approval, whereas others are in clinical trials. Recently, the U.S. Food and Drug Administration (FDA) approved cefiderocol for complex urinary tract infections and Eravacycline for intra-abdominal infections caused by gram-negative microorganisms, including *P. aeruginosa* [19]. Despite ongoing efforts to develop new antibiotics, the pharmaceutical pipeline has struggled to keep pace with the rise of resistance [20].

Recently, secondary plant metabolites have been used to manage MDR pathogens. A study reported that compounds such as neral, 1,8-cineole, and α-curcumene from *Zingiber officinale* essential oil demonstrated strong antibacterial activity against 18 resistant pathogens like *Serratia marcescens*, and carbapenem- and polymyxin-resistant *Klebsiella pneumoniae* [21]. Promising tactics against MDR bacteria involve nanotechnology, such as the use of nanoparticles (NPs) to improve drug diffusion, enhance drug affinity, and increase drug transport. Antibiotics combined with NPs can target medications more effectively. The increased antibacterial activity of metal and metal oxide-based nanoparticles [22] is attributed to oxidative stress caused by reactive oxygen species (ROS), inhibition of biofilm formation, direct bacterial contact, activation of the immune system, and interactions with proteins and DNA [23]. Green-synthesised monodispersed silver nanoparticles [24] nanoparticles showed strong activity against MRSA strains. In a recent study, N′-((5-nitrofuran-2-yl) methylene)−2-benzhydrazide (5-NFB), a novel antibiotic, was delivered using chitosan nanoparticles. The results indicated that drug encapsulation with NPs had improved antibacterial activity against all *S. aureus* strains and had a synergistic effect with the antibiotic [25].

The ability of bacteria to rapidly acquire and spread resistance genes through horizontal gene transfer worsens the crisis. The misuse and overuse of antibiotics are critical factors driving the rise of antimicrobial resistance [26]. In response to these challenges, the scientific community has focused on alternative treatments. Among the most promising are bacteriophages (phages)—viruses that specifically infect and kill bacteria [27]—and CRISPR-Cas systems—bacterial immune tools that have been adapted for gene editing. Phage therapy provides benefits such as high specificity, adaptability, and minimal disruption to the host microbiota, whileCRISPR-Cas technologies enable precise targeting and disruption of resistance genes or vital bacterial functions [28]. The CRISPR-Cas system can be delivered introduced into target bacteria through different methods, with phage-based vectors noted as an effective approach [29].

This review focuses on the combined application of bacteriophages and CRISPR-Cas systems as a novel, synergistic approach to fight MDR infections. By including background information into this introduction, we aim to direct the main text toward recent developments, clinical prospects, and challenges of this promising therapeutic strategy.

## CRISPR-Cas: a precision tool for genome editing

The CRISPR-Cas system—short for Clustered Regularly Interspaced Short Palindromic Repeats and CRISPR-associated proteins—was initially discovered as a bacterial immune mechanism against viral infection. In its naturalsetting, it enables bacteria to detect and neutralize invading phage DNA by incorporating short sequences from past infections into their own genome. These sequences, called spacers, act as a molecular memory, enabling bacteria to respond quickly and specifically to reinfections [30]. Table 2 shows how the CRISPR-Cas systems are categorized for use in antibacterial therapy.

**Table 2:** Classification of CRISPR-Cas systems used in antibacterial therapy

Class	Type	Signature protein(s)	Effector composition	Target molecule(s)	Mechanism of action in antibacterial context	References
Class 1	Type I	Cas3	Multi-protein complex	DNA	Effectively breaks down plasmids that carry resistance genes	[31, 32]
Class 1	Type III	Cas10	Multi-protein complex	DNA and RNA	Cuts DNA and RNA targets; targeting relies on transcription	[31, 33]
Class 1	Type IV	Cas1	Multi-protein complex	DNA	Detect the invasive DNA, break it apart, and then correctly reinsert it at the leader-proximal end of the CRISPR array	[31, 34]
Class 2	Type II	Cas9	Single-protein	DNA	It does not show trans-cleavage activity; it only cleaves the target DNA	[35, 36]
Class 2	Type V	Cas12	Single- protein	DNA	Exhibits trans-cleavage activity by nonspecifically cleaving ssDNA after binding to its target DNA	[35, 36]
Class 2	Type VI	Cas13	Single- protein	RNA	Clear the target RNA and demonstrate collateral activity, where it nonspecifically cleaves other nearby ssRNA	[35, 36]

This bacterial defense mechanism has been transformed into one of the most powerful tools for genome editing. The CRISPR-Cas system has revolutionized genome editing, enabling multiplexed and precise targeting of genetic sequences [37, 38]. The most well-known application is the CRISPR-Cas9 system, where the Cas9 enzyme, guided by a synthetic single-guide RNA (sgRNA), makes precise cuts in target DNA sequences. Its programmable targeting enables highly specific editing across various organisms, including pathogenic bacteria [39]. In combating MDR bacteria, CRISPR-Cas provides a ground breaking approach to directly disrupt antibiotic resistance genes. Many resistance traits are carried on plasmids or other mobile genetic elements which are easily transferred between strains or even species. CRISPR-Cas systems can be engineered to recognize and cut these resistance elements, either removing them entirely or disabling their functional regions [40]. For example, CRISPR has been used to target the blaNDM-1 gene, which is responsible for carbapenem resistance, restoring susceptibility to last-resort antibiotics in clinical isolates.

CRISPR can also be used to induce bactericidal effects by targeting essential genes. This approach turns CRISPR into a selective antibacterial agent, where specificity depends not only on bacterial species but also by genetic content. Importantly, this technique reduces the off-target effects on the host microbiota, which are common with broad-spectrum antibiotics [41]. One of the most innovative applications involves packaging CRISPR-Cas constructs inside bacteriophages. These CRISPR-equipped phages can infect target bacteria and deliver CRISPR payloads directly into the bacterial cytoplasm. Once inside, the CRISPR system activates and cuts the resistance gene, causing cell death or loss of resistance. This dual-use strategy, combining phage infection with genome editing, marks a significant advance in precision antimicrobials [42].

The application of CRISPR to directly modify the phage genome is an active research area. Researchers have reengineered phages to overcome bacterial defense systems such as restriction-modification or anti-phage CRISPR arrays, thereby enhancing phage infectivity and effictiveness. These modified phages have yielded better outcomes in animal infection models, demonstrating real-world potential [43]. Additionally, CRISPR systems can be used to control bacterial populations in complex microbial communities. By selectively targeting harmful strains and preserving beneficial ones, CRISPR enables microbiome manipulation that antibiotics cannot. This approach not only advances infection treatment but also opens new possibilities for microbiome engineering related to gut health, agriculture, and environmental cleanup. Despite its potential, several challenges still exits. Delivery remains one of the biggest obstacles—getting CRISPR components to the targeted bacteria, especially in vivo and within biofilms, demands advanced vectors or carriers. Additional concerns also include off-target effects, immune responses to Cas proteins, and horizontal gene transfer of CRISPR elements, all of which need careful oversight in clinical applications [30]. Horizontal gene transfer, facilitated by mobile genetic elements like integrons and transposons, plays a significant role in spreading resistance [44]. Ethically, the using CRISPR-based antimicrobials in open environment raises questions about potential ecological impacts. While CRISPR’s high specificity is advantageous, incomplete targeting or resistance mutations could lead to escape variants, necessitating biosafety and monitoring systems.

In summary, CRISPR-Cas technology is a transformative addition to the antimicrobial toolkitarsenal. Its precision, flexibility, and adaptability make it an excellent candidate for combination with bacteriophage therapy in the fight against MDR pathogens. Ongoing research into delivery systems, phage engineering, and regulatory approval will influence how quickly and broadly CRISPR-Cas moves from laboratory innovation to clinical use.

## Combining bacteriophages and CRISPR-Cas: synergistic potential

The rise of multidrug-resistant (MDR) bacteria has prompted researchers to look for innovative solutions to combat these dangerous pathogens. One promising approach gaining attention is combining bacteriophage therapy with CRISPR-Cas system. While each technologies has its own benefits when used separately, but together they present a synergistic approach that could revolutionize the treatment of MDR infections. This section explores integrating bacteriophages and CRISPR-Cas, including their benefits, recent advancements, and the challenges faced in implementing this dual strategy.

Bacteriophages are viruses that specifically infect and kill bacteria, while CRISPR-Cas is a precise genome-editing tool that can target and modify bacterial DNA. When combined, these technologies can potentially enhance each other’s effectiveness. Phages are highly specific to their bacterial hosts, allowing them to target and eliminate harmful bacteria without affecting beneficial microbes in the human microbiota. Enzymatic degradation of antibiotics, particularly by β-lactamases, is another key resistance mechanism [45]. However, bacteria can develop resistance to phages just as they do to antibiotics, limiting the efficacy of phage therapy. CRISPR-Cas can be used to modify phages or bacteria to bypass these resistance pathways.. Furthermore, alterations in antibiotic targets, such as changes in ribosomal binding sites or DNA gyrase mutations, play a significant role in resistance [46]. IntegratingCRISPR-Cas into phage therapy enables the creation of phages better equipped to overcoming bacterial defenses, resulting in more effective treatment.

Combination bacteriophages and CRISPR-Cas offers several key advantages. Primarily, CRISPR enhances the effectiveness of phage therapy by enabling precise modifications to both the phages and their bacterial targets. This include editing the phage genome to boost resistance against bacterial defences. For example, CRISPR can alter phage receptor-binding proteins, enabling infection of bacteria that have evolved resistance to initial attacks [47]. Additionally, CRISPR can disable bacterial defense mechanisms, such as the bacterial CRISPR-Cas systems that interfere with phage replication [48].

A major advantage of combining bacteriophages with CRISPR is the ability to minimize collateral damage to the host microbiota. Unlike antibiotics, which can harm beneficial bacteria, phages are highly selective. When used with CRISPR, their combinedprecision targets only the harmful bacteria, preserving the rest of the microbiota and reducing the risk of side effects. This targeted method is especially important for treating infections, such as those in the gastrointestinal tract or on the skin, where maintaining a healthy microbiome is crucial.Furthermore, CRISPR-Cas provides the potential to combat bacterial resistance to phages. Efflux pumps plays a vital role in conferring multidrug resistance by actively extruding antibiotics out of bacterial cells [49]. As mentioned, bacteria can quickly develop resistance to phages by modifying the receptors used for entry or by creating strategies to block phage replication. By incorporating CRISPR-Cas, phages can be engineered to rapidly adapt to these bacterial changes, preventing the development of resistance and ensuring sustained therapeutic efficacy.

Recent studies highlighted the potential of combining bacteriophages and CRISPR-Cas to combat MDR bacteria. Table 3 highlights the main studies that combine bacteriophages and CRISPR-Cas systems to target multidrug-resistant bacteria.

**Table 3:** Summary of key research on using bacteriophages and CRISPR-Cas systems to combat resistant bacteria

Target bacteria	Therapy/approach	Key findings	References
*P. aeruginosa*	Engineered phage with CRISPR-Cas	Reduce the bacteria’s defenses against phage infections, thereby increasing the effectiveness of phage therapy	[50]
*Escherichia coli* strains resistant to colistin	CRISPR-enhanced phages	Significant reduction in bacterial levels seen in animal models	[2]
*E. coli*	CRISPR-Cas9 and Cas3 augmented phages	Completed Phase 1 trial; deemed safe	[51]
*E. coli*	Engineered phage with CRISPR-Cas armament	Promoting effective biofilm penetration helps reduce the development of phage-tolerant *E. coli*	[52]
Kanamycin-resistant *E. coli*	Phage-delivered CRISPR-Cas9	The strategy demonstrated superior antimicrobial activity than lytic phage alone in vitro and in vivo mouse skin and intestinal infection models	[53]
*E. coli* Streptomycin resistant	Phage loaded with pCas9‐GFPT‐f1A/B	Oral administration of phage loaded with CRISPR‐Cas9 phagemid for targeting specific gut microbiome genes; A proof of concept for in vivo targeting strain-specific organisms	[54]
A human isolated *S. aureus* strain	Programmed CRISPR-Cas9 system in noncoding phage genome region	Phage mitigatessoft tissue infection, not bone infection	[55]
*P. aeruginosa*	Anti-CRISPR gene (AcrIF1, AcrIF2, and AcrIF3) -containing phages (EATPs, eat Pseudomonas)	Antibiotic resistance in clinical MDR strains is suppressed; the treatment is safe and effective both in vitro and in vivo	[56]
*Clostridioides difficile*	Temperate phage combined with CRISPR-Cas3	Bacterial clearance related to nuclease activity	[57]
*Klebsiella pnueumoniae*	PhilKpS2 phage with CRISPR-Cas9	Neutralizing MDR genes through gene editing	[58]

These studies demonstrate that combining CRISPR-Cas and bacteriophage therapy is a viable approach to combat highly resistant bacterial strains. Another notable study by [59] successfully engineered phages capable of targeting antibiotic-resistant bacteria and delivering CRISPR-Cas9 systems to directly modify bacterial genomes. This dual-functionality allowed the phages to both kill the bacteria and edit their resistance genes, further enhancing the therapeutic potential of phage therapy.

**Fig. 1:**
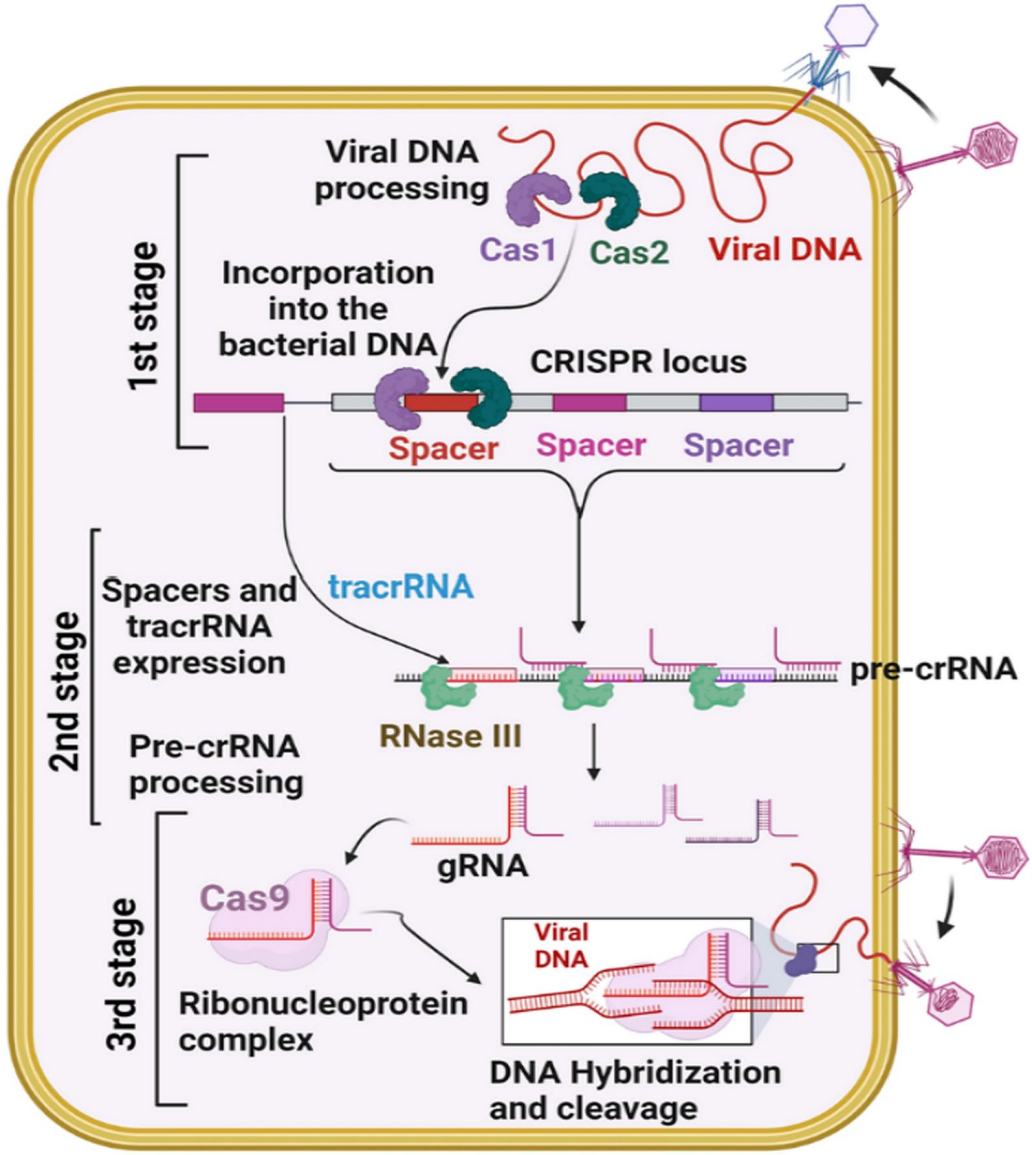
illustrates how a CRISPR-Cas system encoded within a bacteriophage genome is delivered to an intracellular pathogen. The bacteriophages are enclosed in silicon particles, called “cargoes,” which are designed to facilitate entry into the target cell. After entering an infected cell, the bacteriophages target the intracellular pathogen and deliver the CRISPR-Cas system to enable precise genome editing [62]

Although combining bacteriophages and CRISPR-Cas shows promising potential, several challenges remain before clinical application. One major obstacle is effectively delivering both phages and CRISPR components to target bacteria. In order to achieve therapeutic success, the phages and CRISPR-Cas systems must reach the target bacteria efficiently. This is especially difficult with biofilm-associated infections, where an extracellular matrix shield bacteria and blocks the penetration of both agents. Researchers are exploring various delivery strategies, such as using nanoparticles or engineered bacteriophages capable of penetrating biofilms, but effective solutions are still under development [60]. Recent studies have highlighted promising approaches, including engineered temperate phages, liposome-packaged CRISPR systems, and hybrid nanoparticle vectors that improve stability and targeting [61]. Such approaches are essential for reaching intracellular pathogens or bacteria within biofilms (Fig. 1).

Safety and regulatory concerns are also major hurdles. The use of genetically modified organisms (GMOs), including engineered phages or CRISPR-Cas components, raises questions regarding potential off-target effects, unintended genetic modifications, and the long-term impact on the microbiota. As a result, thorough safety evaluations and clinical trials are needed to confirm that these combined therapies are safe and effective for human use. Furthermore, regulatory frameworks for the approving phage therapy and CRISPR-based treatments are still under development, and international regulatory consistency will be crucial to enable widespread adaptation of these therapies [63]. Lastly, the high cost of developing and producing CRISPR-engineered phages at scale could limit widespread use. Although CRISPR-Cas systems have simplified genome editing, creating genetically modified phages and building the infrastructure for clinical application will require substantial investment.

In conclusion, combining bacteriophages with CRISPR-Cas systems offers a synergistic approach with great potential in combat MDR bacteria. This method enhances phages efficiency and overcoming bacterial resistance, making it a promising alternative to traditional antibiotics. However, significant challenges remain in terms of delivery, safety, regulatory approval, and production costs. Despite these hurdles, recent studies have shown that this approach holds great promise for the future of antimicrobial therapy and could play a pivotal role in combating antibiotic-resistant infections.

## Therapeutic applications and clinical prospects

Bacteriophage therapy, once considered outdated due to the advent of antibiotics, has seen a resurgence in recent years due to its precision and adaptability. When combined with CRISPR-Cas, phage therapy can be significantly enhanced to overcome bacterial resistance mechanisms and improve therapeutic outcomes. The synergistic potential of these technologies offers numerous therapeutic opportunities for fighting against MDR bacteria. A primary applications of phage-CRISPR therapy is treating bacterial infections caused by antibiotic-resistant pathogens. Phage therapy offers a natural and targeted way to control bacteria, and it can be engineered to broaden its host range or enhance its lytic capabilities [50, 64]. Phage therapy can specifically target resistant bacteria, while CRISPR-Cas can be used to disrupt bacterial resistance genes or enhance the efficiency. For example, CRISPR-Cas can be used to modify phage genomes, enabling them to bind more effectively to bacterial cells that have evolved resistance [30, 47]. Furthermore, CRISPR-Cas systems can target and inactivate bacterial defense mechanisms, such as the bacterial CRISPR systems that prevent phage infection, allowing phages to kill bacteria more efficiently.

In chronic infections where bacteria form biofilms that resistantibiotics and phages, the combination of CRISPR and phages hold great promise. These biofilm-associated infections are complicated to treat because they shield bacteria from immune responses and medications. However, studies have shown that CRISPR can be used to target the biofilm matrix, reducing bacteria’s resistance to phage infection [65, 66]. This approach could significantly improve the effectiveness of phage therapy in ln term and biofilm-related infections, such as those connected to medical devices, chronic wounds, and cystic fibrosis. Furthermore, phage-CRISPR therapy has potential for gastrointestinal infections caused by antibiotic-resistant *Clostridium difficile* or *Escherichia coli*. Phages, can specifically attack bacteria, while CRISPR-Cas, which can edit or disable resistance genes, potentially transforming treatment of these often-life-threatening infections. Furthermore, CRISPR-Cas technology can be used to engineer phages that not only kill resistant bacteria but also modify their genomes, potentially preventing future resistance [40].

Although the combination of bacteriophages and CRISPR-Cas is still in the early stages of development, several pilot studies and clinical trials evaluating their effectiveness and safety. In 2019, a team of researchers in the United States conducted a clinical trial using phage therapy with CRISPR-Cas9 to treat *P. aeruginosa* infections in a patient with cystic fibrosis. The trial showed a decrease in bacterial load and symptoms improvement, marking an important step toward clinical application [49]. This trial was among the first to explore the integration of CRISPR into phage therapy for the treatment of chronic infections, highlighting its promise as an additional treatment.

In a 2020 study, a research group in Europe successfully demonstrated the use of CRISPR-modified bacteriophages in animal models to treat *Escherichia coli* infections resistant to colistin, a last-resort antibiotic. By editing the phage genome using CRISPR-Cas9 to overcome resistance mechanisms, the researchers achieved a significant reduction in bacterial load and better therapeutic outcome than with the traditional phage therapy [42]. This provided important proof that CRISPR-enhanced phages can effectively treat MDR bacterial infections in vivo. While clinical trials are still limited, these early successes suggest that phage-CRISPR therapies have the potential to become a promising alternative to antibiotics, particularly for treating infections caused by highly resistant bacteria. The next step is to expand these trials to include more pathogens and diverse patient groups toevaluate the safety, efficacy, and long-term effects..Although promising, several challenges remain before widespread clinical use. Developing effective delivery systems remains a major hurdle; both phages and CRISPR-Cas systems must reach infection site remain active for long enough to produce therapeutic effects, which is particularly challenging in deep tissue infections, biofilm-associated infections, and in patients with compromised immune systems. Researchers are exploring various delivery strategies, including nanoparticles and viral vectors, to protect both phages and CRISPR components during delivery and remain stabilized during delivery [37, 38, 48].

To enhance stability and in vivo delivery precise, CRISPR components are encapsulated within nanoscale carriers like virus-like particles (VLPs) [67]. These VLPs carry gene editing tools, such as plasmid DNA, mRNA, or ribonucleoproteins, mimicking viral infection to enter target cells [68]. Techniques such as electroporation and microinjection deliver CRISPR directly into targeted cells, offering high precision but are sometimes limited to ex vivo use due to their invasive nature [69]. Artificial Intelligence (AI) rapidly transforms CRISPR/Cas9 research by improving guide RNA design using algorithms that ensure precise targeting and reduce off-target effects. These algorithms analyze large datasets to identify the best gRNA sequences for various applications. AI also enhances delivery methods by helping select the most effective vectors and techniques. Its use accelerates development and improves the safety and reliability of CRISPR/Cas9 treatments [70]. The controlled release of phage therapies can be achieved through encapsulation in biomaterials. Embedding phage-loaded biopolymers into surgical implants or during procedures may reduce the need for repeated doses if phage stability is maintained. Studied biopolymers include hydrogels from collagen, fibrin, agarose, and alginate, along with synthetic polymers such as polyethylene glycol (PEG), polyacrylamide (PA), and polyvinyl alcohol (PVA) [71]. Bacteriophages, with polyvalent ligands, serve as scaffolds [72] and have numerous applications, including diseases through various delivery systems and routes, environmental monitoring of pathogenic bacteria, and delivering bioactive agents for targeted therapeutic benefits [73].

Regulatory hurdles also present a significant obstacle to the clinical use of phage-CRISPR therapies. While CRISPR technology is already used in gene therapy and other medical fields, its application in human treatment remains highly regulated. Additionally, phage therapy, especially when combined with CRISPR, involves genetically modified organisms, which raises safety and ethical concerns. Extensive clinical trials are necessary to ensure the safety these therapies are safe and to obtain regulatory approval from agencies such as the FDA and EMA. Furthermore, new regulatory guidelines are needed to oversee the production, testing, and application of phage-CRISPR therapies. Despite these challenges, the potential benefits of combining bacteriophages and CRISPR-Cas systems are immense. The ability to precisely target and modify bacterial genomes could revolutionize the treatment of MDR infections and reduce the reliance on antibiotics. Moreover, by minimizing collateral damage to the microbiota and overcoming bacterial resistance mechanisms, phage-CRISPR therapies could be a breakthrough in fighting antibiotic-resistant bacteria.

To effectively address antibiotic resistance in the future, the first crucial step is to raise awareness through education, persuasion, and advocacy. However, we must acknowledge our limitations and strive to understand the causes and mechanisms behind the problem. Conventional methods are no longer sufficient. Innovative ideas, tactics, and techniques are necessary to address challenges that could contribute to the development of AMR. Key needs include advanced diagnostic tools, automation, and financial support to help combat resistance. Additionally, revitalizing antimicrobial drug use by exploring new method could help decrease resistance [41].

Research on drug-resistant bacteria explores innovative strategies to overcome resistance and develop alternative therapies. Progress includes next-generation antibiotics and synthetic analogues, such as synthetic teixobactins, which target multiple essential lipids in bacteria like MRSA, making resistance difficult [74]. CRISPR/Cas9 can target bacterial genes or resistance plasmids, enhancingsensitivity or destroying specific strains [75]. Pathogen-focused methods target particular bacteria, while gene-focused approaches target resistance genes on plasmids; their effectiveness varies [76]. Nanoparticles can disrupt membranes and biofilms, offering alternatives for antibiotic-free or coated treatments, especially when combined with natural compounds, though toxicity concerns remain [77]. AI and machine learning (ML) technologies are advancing the detection of resistance. AI helps analyze large biological datasets to quickly and accurately predict bacterial antibiotic sensitivity and resistance, supporting antimicrobial decisions and disease management [78]. These Technologies have also uncovered new resistance mechanisms and introduced innovative strategies to optimize antibiotic use [79].

Phage-CRISPR therapies stand out from other antimicrobial methods due to their combined use of naturally targeting phages and the programmable genetic capabilities of CRISPR-Cas systems. The lack of species specificity of traditional antibiotics is a significant drawback, as they may disrupt the metabolism and structure of bacterial communities that contain both pathogenic and beneficial strains. By allowing targeted disruption of specific bacterial DNA sequences (as opposed to viral ones), this dual approach not only makes it easier to eradicate bacteria by phage-induced lysis, but also increases the efficacy of treatment [80]. Additionally, the coevolution of phages and bacteria [81] and the adaptability of CRISPR sequence significantly slow the development of resistance [82]. Bacterial biofilms pose a significant challenge and are often responsible for the failure of antibiotic treatments for various hospital infections. To combat biofilms, phage engineering has become a key tool for the targeted improvement of certain phage properties [83]. Personalised treatment and a genomics-based approach are poised to transform the management of bacterial infections. Modern treatment options can incorporate targeted treatments or antibiotics optimized based on analysis of lifestyle choices, genetic factors, and environmental factors [84]. Antimicrobial peptides can affect a wide range of microorganisms, but they may also damage beneficial microbiota[85]. Phage-CRISPR therapies provide high specificity by using phages to target particular bacterial strains and CRISPR sequences for sequence-level precision, sparing non-target species [86, 87]. Concerns about the environmental impact of nanoparticles’ (NPs) are growing, alongside concern about their health effects. Due to their small size and unique properties, NPs can affect various components of the natural ecosystem, potentially disrupting ecological balance, accumulating in the food chain, and impacting species, posing a risk to both humans and wildlife. For example, research on materials such as titanium dioxide nanoparticles (TiO_2_ NPs) and silver nanoparticles (Ag NPs) has demonstrated their toxicity to both aquatic and terrestrial animals, leading to ecosystem disturbance and biodiversity loss [88]. Phage therapy offers a promising option for treating infections caused by bacteria that are sensitive to or resistant to antibiotics, with minimal side effects on patients [89].

In the coming years, we can expect continued research and clinical trials to refine phage-CRISPR therapies, improve delivery methods, and address regulatory and safety concerns. With ongoing advancements in biotechnology, phage-CRISPR therapies could soon transition from laboratory studies to routine clinical practice, offering a new hope for patients suffering from MDR bacterial infections.

## Challenges and limitations

Despite the promising potential of combining bacteriophages and CRISPR-Cas systems to combat multidrug-resistant (MDR) bacteria, several scientific, technical, regulatory, and societal challenges must be addressed before these therapies can be widely adopted. These challenges include bacterial resistance to phages andethical and regulatory concerns related to gene editing technologies. In this section, we explore the main obstacles in the development and clinical application of phage-CRISPR therapies.

One of the major scientific challenges is the development of resistance to bacteriophages. Just as bacteria evolve resistance to antibiotics, they can also find ways to avoid phage infection. Phage resistance can occur through various mechanisms, such as changes to bacterial surface receptors that phage attach to, activation of bacterial defense systems like CRISPR-Cas, or the alteration of bacterial enzymes that break down phage genomes [50, 90]. This resistance can greatly reduce the effectiveness of phage therapy, necessitating the constant development of new phages or phage combinations to overcome resistance.

Beside phage resistance, delivery challenges pose a significant obstacle. Both bacteriophages and CRISPR-Cas components must be delivered to bacterial cells within the body, especially in deep tissues or biofilms. While phages can be applied directly to the site of infection, ensuring they reach the target bacteria in sufficient concentrations without being cleared by the immune system remains difficult. Similarly, CRISPR-Cas systems require precise delivery methods to prevent off-target genetic modifications or immune responses. Recent progress in nanoparticles, viral vectors, and liposomes have shown promise as delivery vehicles, but further research is required to optimize these methods for clinical use [46, 60].

Off-target effects, another technical issue, are especially important when using CRISPR-Cas systems. Although CRISPR-Cas9 is known for its precision, it still presents risks. The chance of accidental gene editing can lead to undesirable consequences, such as the disruption of essential bacterial functions or causing harmful mutations in the host microbiota. Efforts are being made to improve the specificity of CRISPR systems, including the development of high-fidelity Cas9 variants and other Cas enzymes that minimize off-target edits [45, 62].

The integration of CRISPR-Cas systems and bacteriophage therapy raises several regulatory and ethical questions. CRISPR technology, while innovative, remains tightly regulated due to its gene-editing capabilities. Specifically, the use of CRISPR for human is under significant scrutiny. Ethical concerns related to gene editing, especially in humans, include the risk for unintended genetic consequences, misuse of the technology for non-therapeutic purposes, and the risk of genetic modifications being passed on to future generations. Agencies, like U.S. Food and Drug Administration (FDA) and the European Medicines Agency (EMA), need to establish clear guidelines and safety standards for CRISPR-based therapies, ensuring they are thoroughly tested for safety and effectiveness before clinical application [30, 66].

Phage therapy faces regulatory challenges. Although phages are naturally occurring and have been historically used to treat bacterial infections, the use of engineered phages or phage-CRISPR combinations requires thorough safety assessments. The risk for phages to unintentionally transfering genes, including antibiotic resistance genes, to bacteria or disruptions the host microbiota raises safety concerns [89]. Regulatory frameworks must adapt to these new biotechnologies, establishing clear pathway for clinical approval while ensuring safety. Public perception and acceptance of phage therapy and gene editing technologies also play crucial role in their widespread adoption. While phage therapy has a long history in some parts of the world, particularly in Eastern Europe and the former Soviet Union, it remains relatively unfamiliar in Western medicine. The lack of public awareness about phage therapy and the complexities of gene editing may hinder its acceptance, particularly when compared to traditional antibiotic treatments, which are more widely understood and trusted. Overall, public perception of both phage therapy and gene editing technologies remains cautious. A survey by [60, 62] suggests that while interest in phage therapy is growing, concerns about genetic modification linger, especially in areas where CRISPR remains ethically controversial. Transparent education and clinical success stories can help build public trust and acceptance.

Gene editing, especially CRISPR technology, also faces public concern, mainly due to its potential for misuse in areas such as human germline editing or genetic enhancement. While the application of CRISPR to treat bacterial infections may be seen as less controversial, concerns about long-term consequences and unintended outcomes remain. Education and transparent communication regarding the safety, potential benefits, and ethical considerations of phage-CRISPR therapies are crucial for gaining public trust. Combining bacteriophages with CRISPR-Cas systems offers a promising approach to combating MDR bacteria globally. However, significant challenges remain before these therapies can become routine in clinical practice. These include technical issues such as phage resistance, delivery issues, off-target effects, and regulatory and ethical concerns related to gene editing and phage safety. Public perception and acceptance also play a critical role in the successful implementation of these therapies. Continued research, clear communication, and the development of strong regulatory frameworks will be key to overcoming these challenges and unlocking the full therapeutic potential of phage-CRISPR systems.

## Conclusion

The combination of bacteriophages and CRISPR-Cas systems is among the most promising strategies for combatingmultidrug-resistant (MDR) bacterial infections. As antimicrobial resistance continues to grow and outpace the development of new antibiotics, it has become clear that innovative, targeted, and adaptable methods are essential to protect public health. This review examines how combining phage therapy with CRISPR-Cas genome editing offers a synergistic and highly customizable alternative to traditional antibiotics, with a potential that extends well beyond current clinical applications.

By harnessing the natural specificity and bactericidal capacity of phages alongside the precision gene-editing ability of CRISPR-Cas systems, researchers are developing platforms that not only eliminate resistant bacteria but also deactivate resistance genes, reduce off-target effects, and minimize the impact on the host microbiota. This dual-action approach could significantly transform our treatment options, particularly for chronic infections, biofilm-related conditions, and hospital-acquired infections, which are often caused by MDR pathogens.

Emerging research supports the clinical potential of this approach by showing how phages engineered with CRISPR-Cas systems directly into bacterial populations. These engineered phages can selectively target resistance genes, even within heterogeneous microbial communities, offering a level of specificity that traditional antibiotics cannot match. Moreover, CRISPR-enhanced phages can be quickly customized to combat new bacterial threats, making them especially useful for personalized or precision medicine.

Despite promising progress, several hurdles must be addressed before these technologies can be fully integrated into clinical practice. Significant challenges include creating reliable delivery systems that can efficiently transport phages and CRISPR components to infection sites, particularly those involving biofilms or difficult-to-access tissues. There are also concerns about immune responses to bacteriophages and CRISPR-Cas proteins, the potential for unintended genetic changes, and the ecological effects of widespread use. These scientific and ethical issues demand thorough research, transparent reporting, and robust regulatory frameworks.

Regulatory uncertainty remains a critical barrier. Although agencies have begun to oversee phage therapy, combining it with gene editing adds further complexity that current frameworks are not yet ready to handle. Collaboration among scientists, regulatory authorities, and policymakers is essential to developing guidelines that ensure the safe, ethical, and effective. Public engagement is also play an important role, especially given concerns about gene editing technologies. Building public trust through education and responsible communication will be key to broad acceptance.

Looking forward, ongoing interdisciplinary research is essential to refine these technologies, assess their long-term impacts, and identify best practices for clinical use. Investment in scalable manufacturing, standardized protocols, and thorough clinical trials will help bridge the gap between laboratory discovery and real world application. If these challenges are addressed, phage-CRISPR therapies could become vital tools in future antimicrobial strategies, helping to fight the global threat of antibiotic resistance while preserving the effectiveness of current treatments.

Ultimately, the merging of synthetic biology, microbiology, and precision gene editing through bacteriophage and CRISPR-Cas has the potential to transform infectious disease treatment. By targeting bacterial pathogens on both functional and genetic levels, this approach promises a new era of smart, adaptable, and sustainable antimicrobial therapy—helping us tackle one of the most pressing medical challenges today. to overcoming one of the most urgent medical challenges of our time.

## Data Availability

All the data in the manuscript are obtained from included references and available upon request.
